# Games in Times of a Pandemic: Structured Overview of COVID-19 Serious Games

**DOI:** 10.2196/41766

**Published:** 2023-03-07

**Authors:** Tjaša Kermavnar, Valentijn T Visch, Pieter M A Desmet

**Affiliations:** 1 Human-Centered Design Industrial Design Engineering Delft University of Technology Delft Netherlands

**Keywords:** COVID-19, serious game, game-based intervention

## Abstract

**Background:**

The COVID-19 pandemic introduced an urgent need for effective strategies to disseminate crucial knowledge and improve people’s subjective well-being. Complementing more conventional approaches to knowledge dissemination, game-based interventions were developed to create awareness and educate people about the pandemic, hoping to change their attitudes and behavior.

**Objective:**

This study provided an overview and analysis of digital and analog game-based interventions in the context of the COVID-19 pandemic. As major pandemics and other large-scale disruptive events are expected to increase in frequency in the coming decades, this analysis aimed to inform the design, uptake, and effects of similar future interventions.

**Methods:**

From November 2021 to April 2022, Scopus, Google, and YouTube were searched for articles and videos describing COVID-19–themed game-based interventions. Information regarding authorship, year of development or launch, country of origin, license, deployment, genre or type, target audience, player interaction, in-game goal, and intended transfer effects was extracted. Information regarding intervention effectiveness was retrieved where possible.

**Results:**

A diverse assortment of 23 analog and 43 digital serious games was identified, approximately one-third of them (25/66, 38%) through scientific articles. Most of these games were developed by research institutions in 2020 (13/66, 20%) and originated in Europe and North America (38/66, 58%). A limited number (20/66, 30%) were tested on relatively small samples, using a diversity of research methods to assess the potential changes in participants’ knowledge, attitudes, and behaviors as well as their gameplay experience. Although most of the evaluated games (11/20, 55%) effectively engaged and motivated the players, increased awareness, and improved their understanding of COVID-19–related issues, the games’ success in influencing people’s behavior was often unclear or limited.

**Conclusions:**

To increase the impact of similar future interventions aimed at disseminating knowledge and influencing people’s attitudes and behaviors during a large-scale crisis, some considerations are suggested. On the basis of the study results and informed by existing game theories, recommendations are made in relation to game development, deployment, and distribution; game users, design, and use; game design terminology; and effectiveness testing for serious games.

## Introduction

### Background

The outbreak of the novel coronavirus causing COVID-19 was declared a pandemic in March 2020 [1]. With increasing restrictions and uncertainty, a growing number of studies have reported troubling large-scale psychological effects of the COVID-19 pandemic, including worry, fear, anxiety, boredom, frustration, irritability, anger, sadness, and depression [2-7]. On a collective level, health anxiety, collective disorientation, community panic, crisis fatigue, and social isolation fatigue have been observed [3,4,8]. These effects seem to be perpetuated by *doomsurfing* or *doomscrolling*, which refers to the tendency to continually search for bad news on the web even though it is saddening, disheartening, or depressing [9]. An increase in additional dysfunctional behaviors such as panic purchasing of essential household items, increased consumption of psychoactive substances, weight gain because of stress-induced eating, and complete social withdrawal has suggested an imbalance between increased environmental demands and the efficacy of people’s resilience and coping. Experts have advised that people adhere to daily routines and maintain a strong degree of social connectedness to improve communication and reduce boredom [10-12]. Less conventional means to maintain or improve people’s subjective well-being have also been investigated, including digital health interventions (ie, telemedicine, eHealth, and mobile health) [13-15], exergames [16], arts-based interventions [10], bibliotherapy [17], and the company of pet animals [18].

Playing games has been a popular coping strategy during the pandemic [19,20]; it has even been recommended by the World Health Organization (WHO) as an effective way to stop the spread of COVID-19 [21]. Kleinman et al [22] reported that people resorted to games to maintain their mental well-being (eg, to cope with loneliness, escape from the troubling real world, or replace the loss of routines because of quarantine), connect with others (eg, with distanced friends and family, people confined to the same household, or new people), or substitute reality (eg, using game characters as a substitute for real interactions and hosting internet-based events). Some people have also found solace in creating games or gamifying everyday activities to cope with distress during quarantine [22].

During the pandemic, an increased interest specifically in pandemic-themed games such as the board game *Pandemic,* which was developed after the 2003 severe acute respiratory syndrome epidemic, was observed [23]. In 2020, over 50 global game industry leaders launched the campaign *#PlayApartTogether* to encourage gamers to follow the WHO guidelines as well as incorporate COVID-19 prevention messages into their games [24,25]. Thus, in addition to *entertainment* games intended to be played primarily for amusement purposes, a considerable number of *serious* games were developed to educate people about the COVID-19 pandemic, influence their behavior, and improve their well-being in terms of increased life satisfaction and decreased negative affect. These games drew upon the proven positive effect that (serious) games can have on increasing health literacy [26,27]. Although the entertainment in playing games can in itself benefit one’s psychological well-being, our study focuses on *serious* games that were intentionally designed to spread awareness and knowledge of COVID-19, promote healthy behaviors, and increase people’s resilience during the pandemic.

*Serious* games (hereinafter referred to as *games*) often use entertainment qualities to increase player motivation to learn and change their attitudes or behavior in the real world (ie, to facilitate *transfer effects* such as contagion-preventive behavior) [28]. They can increase people’s understanding of complex situations and equip them with the knowledge and skills that are required in real life [24], especially when immersive simulations of threatening new or unusual situations are used in a safe environment that players can explore to prepare for the possible consequences of a real disaster [29]. Although multiple theories on the motivational effects of game and play (starting in 1938 with *Homo Ludens* by Huzinga [30,31]) have been developed, it is very difficult to specify the exact game elements that cause games’ transfer effects [32,33]. Some theorists have stated that games are ideal candidates for optimally responding to universal motivational needs (cf the relationship between games and self-determination theory [34]), whereas other theorists [35] have claimed that games provide a safe space to explore and play along the dimensions of rule-based games (*ludus*) and spontaneous play (*paidia*). In addition to the motivational effects of game elements, such as rewards, challenges, or imagination [36], games can motivate people by offering an alternative world that is fun, safe, and engaging. Huizinga [30] dubbed this (experienced) game world “the magic circle,” defining it as “a temporary world within the ordinary world with specific time- and space-boundaries.” Several theories, such as the theory of narrative transportation [37] and make-believe [38], describe why people tend to be attracted to these alternative worlds. Serious games make use of the motivational aspects of (entertainment) games to intentionally achieve effects in the *ordinary* or *real* world. These can involve effects on awareness (cf health risk awareness [39]), health knowledge [40], health attitudes [41], and health behaviors [42].

### Objectives

Recent reports such as that by Metabiota [43] have warned that the risk of another major pandemic during our lifetimes is higher than many expect—they estimate the likelihood of another pandemic occurring within the next 25 years to be 47%-57%. Similarly, Marani et al [44] predicted up to a 3-fold increase in the yearly probability of extreme epidemics in the coming decades. This implies that the games developed in response to the recent pandemic could potentially serve as a basis for future games, indicating a need to explore the landscape of COVID-19–themed games. Apart from a recent analysis of 5 studies that evaluated 4 games in total [45], to date, no comprehensive reviews that could help evaluate the various game design decisions for this specific context have been published. Thus, the overview presented in this paper aims to inform the design of similar game-based interventions by showcasing the diversity of intentions and design strategies.

## Methods

### Search and Selection of COVID-19 Games

In November 2021, we performed a search of Scopus to identify scientific articles that described the development or testing of COVID-19–themed games. The search was limited to articles and conference papers published in English after 2018 that included the keyword “game*” and any of the keywords “covid*,” “sars-cov*,” “pandemic*,” or “lockdown*” in the title. To further reduce the number of irrelevant results, we excluded those where the word *game* was used in relation to *game changer* or the Olympic and Paralympic games. The search was then repeated in April 2022 to include the most recent studies. Titles and abstracts of the identified 181 results were screened to exclude publications that did not focus on games developed to educate the general public on the COVID-19 pandemic. In total, 34 full texts describing 25 different games were included in the review.

An important consideration that informed the literature review was that games are not necessarily disseminated through or discussed in scientific publications. To avoid excluding relevant games that had only been disseminated through nonscientific media, Google and YouTube were also searched using the aforementioned keywords. This search, which was performed from November 2021 to April 2022, identified 42 additional games developed by private citizens, studios, and profit and nonprofit organizations. Owing to the overwhelming number of prototypes of digital games developed primarily for game jams (eg, International Festival of Independent Games “JAMMING THE CURVE: COVID-19 Game Jam”) or competitions (eg, the 2021 Institute of Electrical and Electronics Engineers Conference on Virtual Reality and 3D User Interfaces contest “Challenging Pandemics”), these were not included in this study.

### Data Extraction

The identified games were analyzed and categorized based on the Comprehensive Taxonomy for Serious Games by De Lope and Medina-Medina [46]. This taxonomy builds on a series of concepts from existing partial classification systems to collect and organize a large number of game features when developing new games or when analyzing or comparing existing ones [46]. Information regarding the games’ authorship, license, deployment, genre or type, target audience, player interaction, in-game goal, and intended transfer effects was extracted. Information regarding the year of development or launch, country of origin, and user testing was also obtained where possible.

## Results

### COVID-19 Games

#### Overview

By January 2022, a total of 66 COVID-19–themed games—23 (35%) analog and 43 (65%) digital—were identified. Detailed overviews of analog and digital games are provided in Multimedia Appendix 1 [47-52] and Multimedia Appendix 2 [53-70], respectively; Figures 1-10 show 10 game examples. The general findings are presented in the following sections.

**Figure 1:**
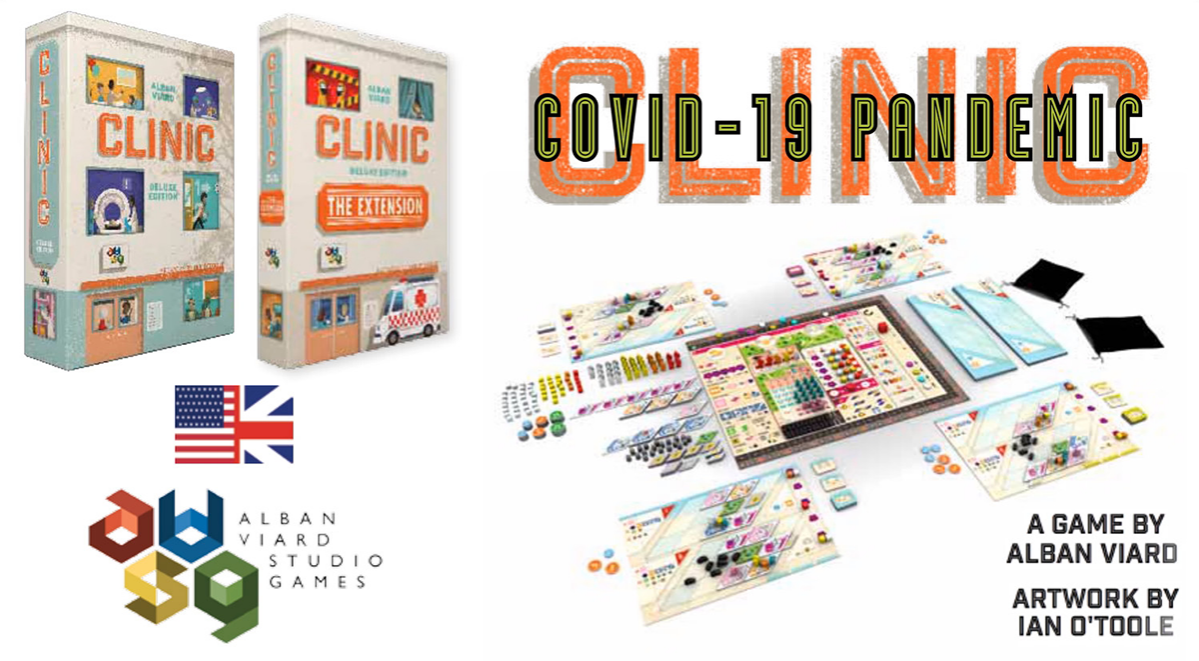
Clinic Deluxe Edition: CoVid_19 variant (image reproduced with permission from Alban Viard [71]).

**Figure 2:**
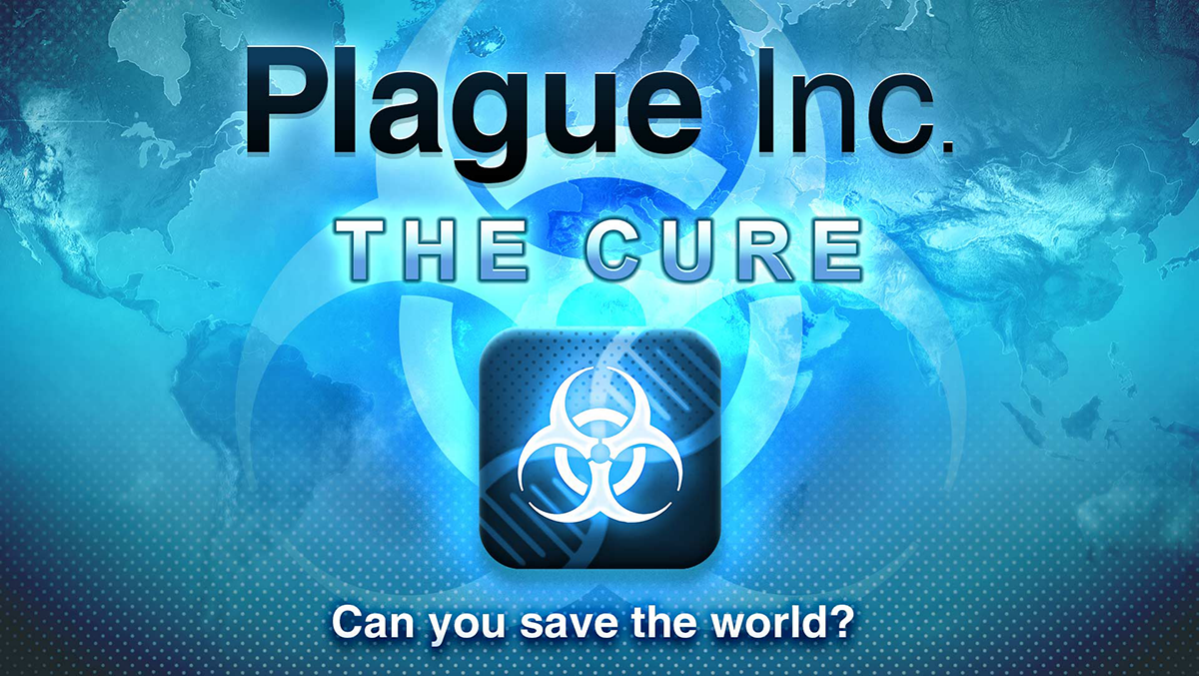
Plague Inc.: The Cure (image reproduced with permission from Ndemic Creations [72]).

**Figure 3:**
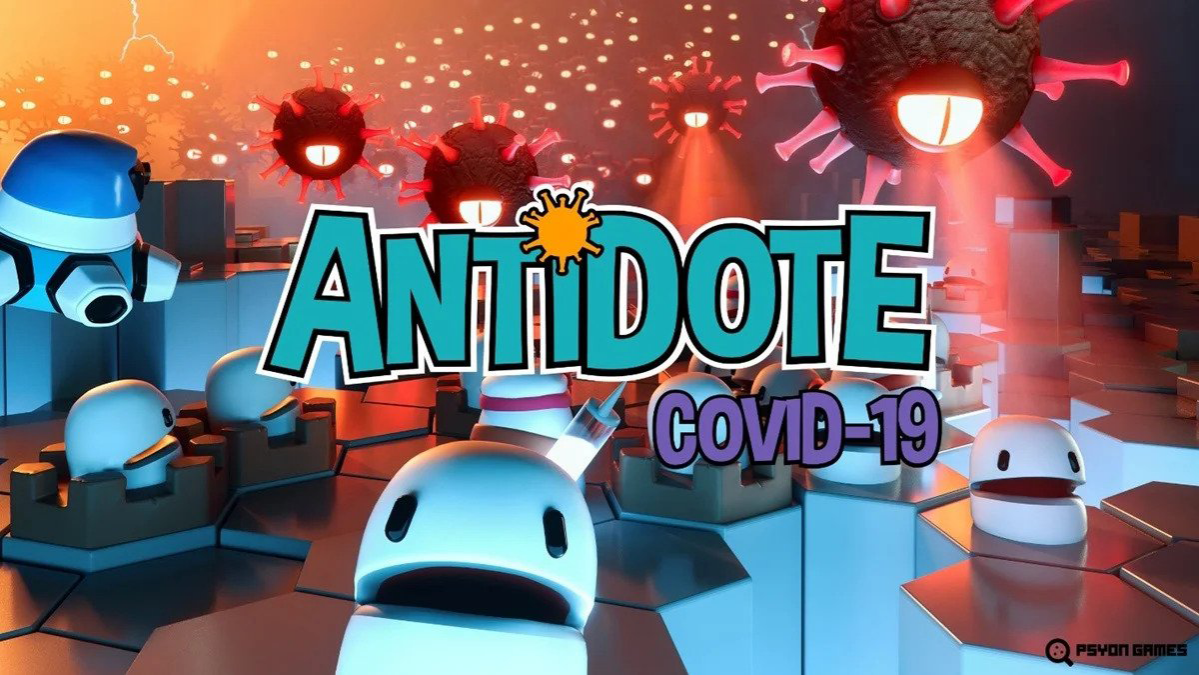
Antidote COVID-19 (image reproduced with permission from Psyon Games [73]).

**Figure 4:**
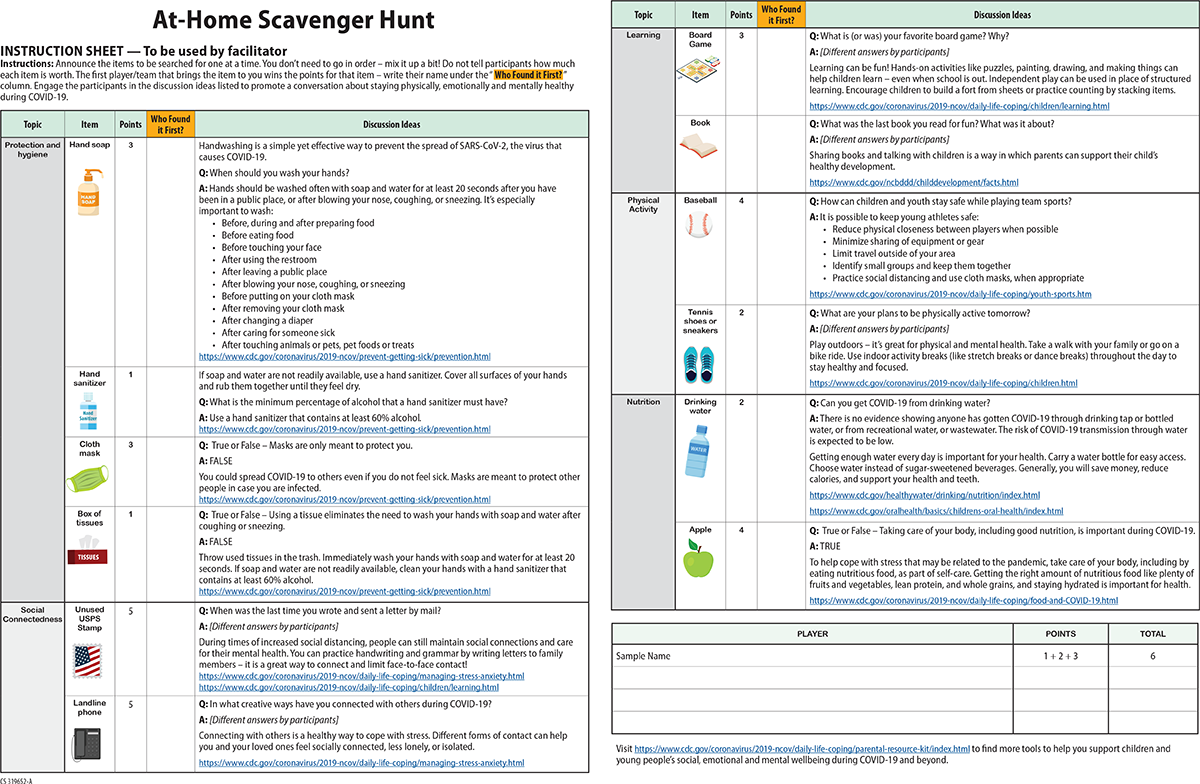
At-Home Scavenger Hunt [47]. Materials developed by the Centers for Disease Control and Prevention (CDC). Reference to specific commercial products, manufacturers, companies, or trademarks does not constitute its endorsement or recommendation by the US Government, Department of Health and Human Services, or Centers for Disease Control and Prevention.

**Figure 5:**
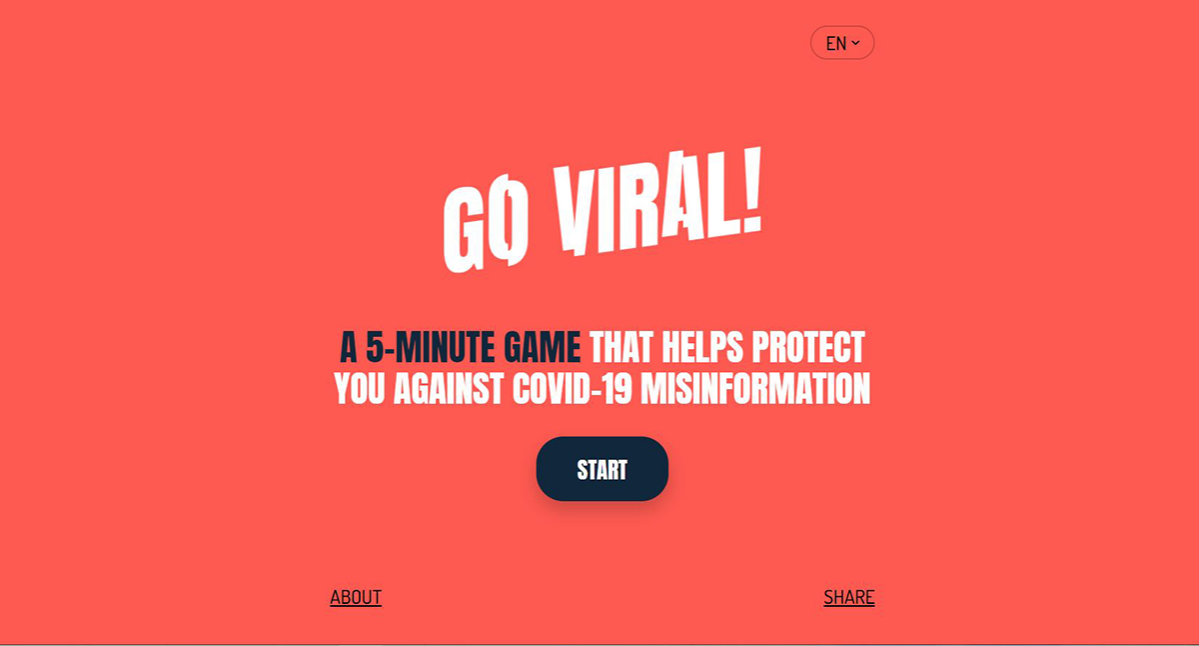
GO VIRAL! (image reproduced with permission from Tilt Studio and Professor Sander L van der Linden from the University of Cambridge [66]).

**Figure 6:**
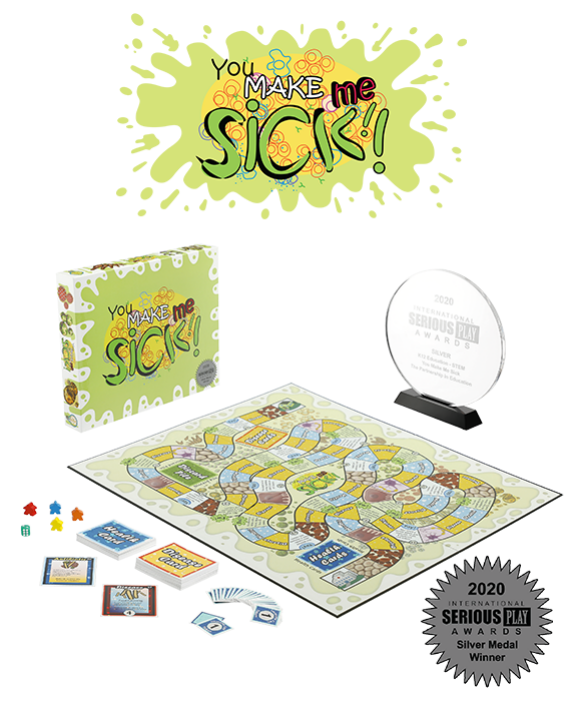
You Make Me Sick! (image reproduced with permission from The Partnership in Education [52]). The game was created by The Partnership in Education, a project supported by the National Institute of General Medical Sciences of the National Institutes of Health under award R25GM132910.

**Figure 7:**
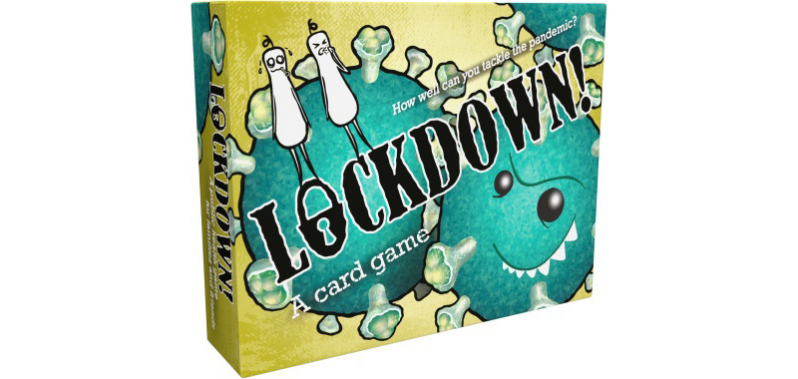
Lockdown! (image reproduced with permission from Yann Boucher [76]).

**Figure 8:**
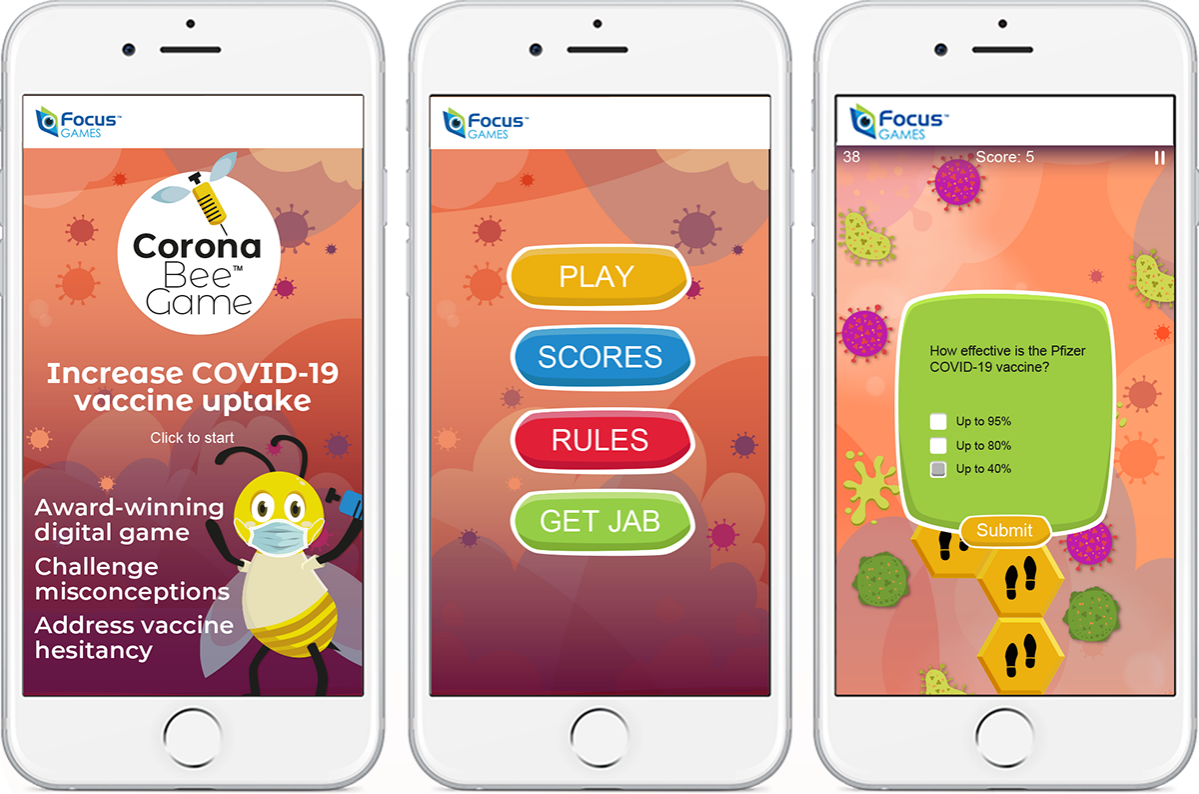
Corona Bee (image reproduced with permission from Focus Games [56]).

**Figure 9:**
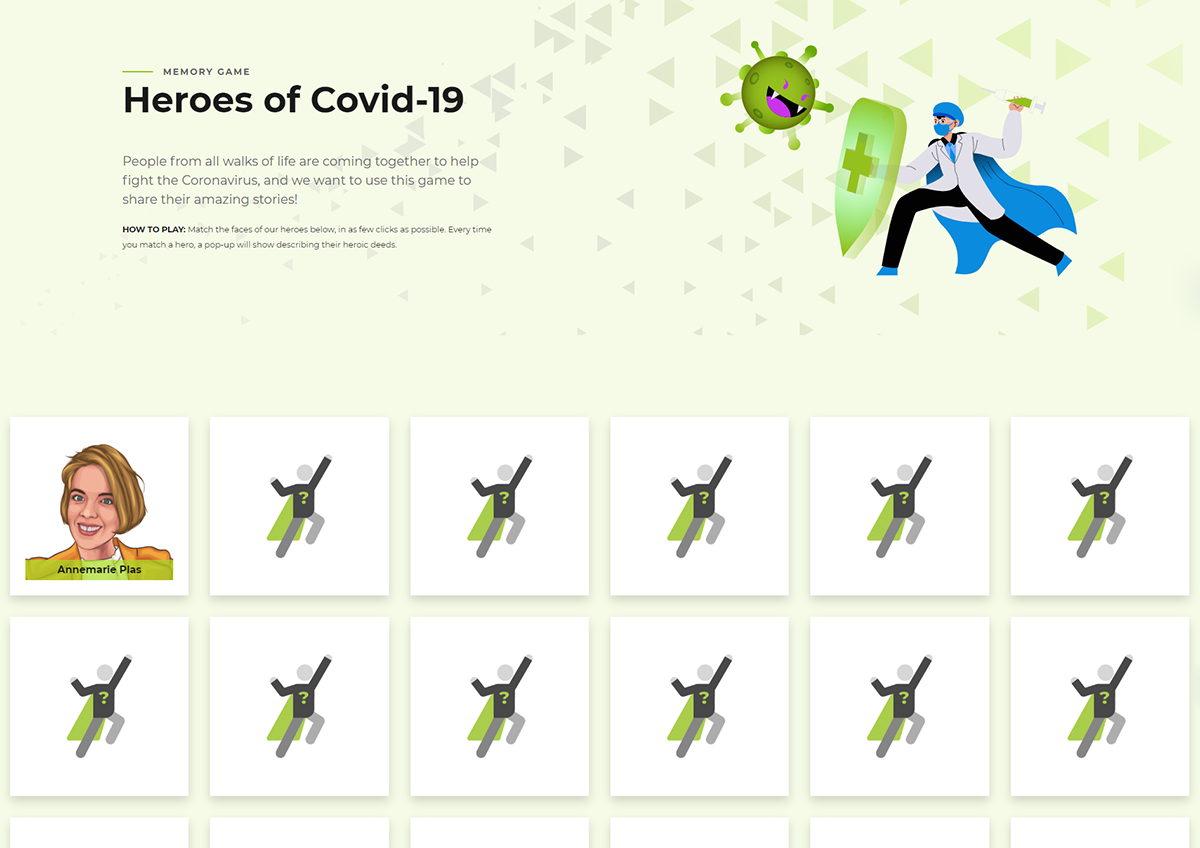
Heroes of Covid-19 (image reproduced with permission from GRM Digital [67]).

**Figure 10:**
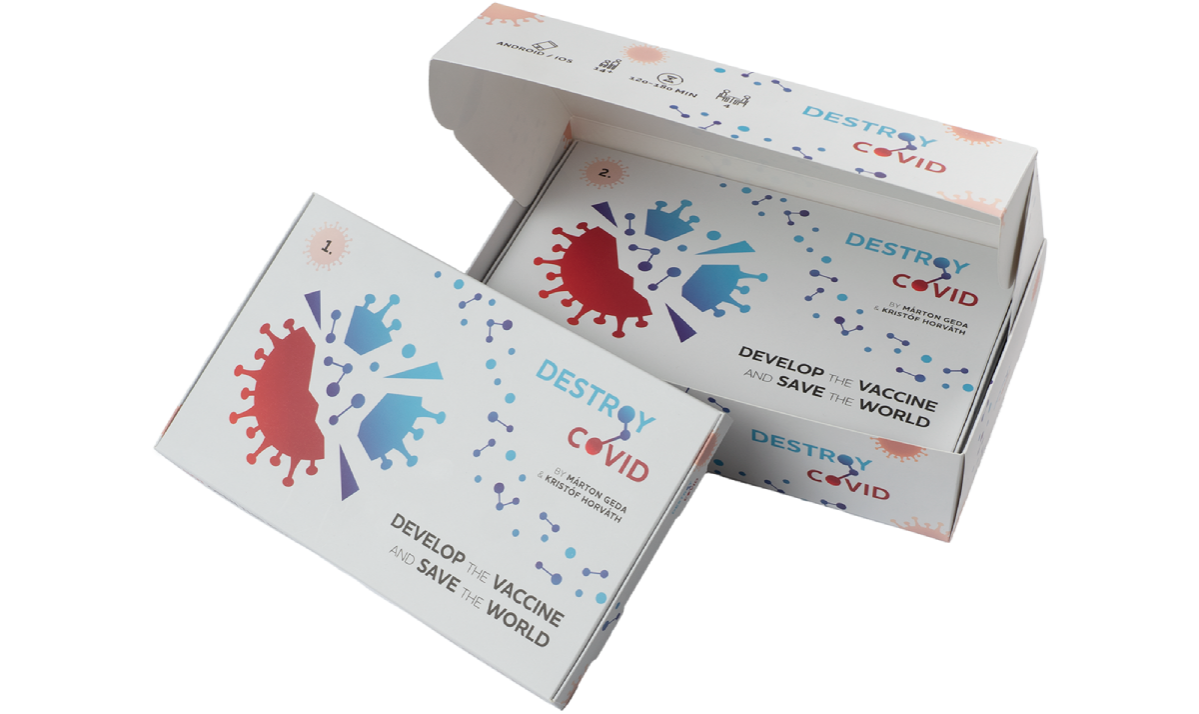
Destroy COVID (image reproduced with permission from Kristóf Horváth [74]).

#### Release Year and Location

The vast majority of the identified games were developed or launched in 2020 (41/66, 62%); the rest were developed or launched in 2021. The largest number of games originated in the United States (10/66, 15%), India (9/66, 14%), and the United Kingdom (7/66, 11%). The countries of origin of other games included Denmark, the Netherlands, and Switzerland (3/66, 5% of the games each); Canada, Czech Republic, Finland, Germany, Morocco, Portugal, and South Africa (2/66, 3% of the games each); and Australia, Bangladesh, Belgium, Brazil, China, France, Hungary, Indonesia, Poland, Saudi Arabia, Singapore, and Taiwan (1/66, 2% of the games each). For 8% (5/66) of the games, the country of origin could not be determined.

#### Authorship

The lead developers were research institutions (31/66, 47% of the games), game development studios (17/66, 26% of the games), or private individuals (13/66, 20% of the games). A total of 9% (6/66) of the games were developed by children aged 10 to 17 years (*Better than Hugo, Corona – Mit Eifer ins Geschäft, Corona Yuga, COVID-19: A Race to the Vaccine, Go Corona Go,* and *Infected!*). Governmental and nongovernmental organizations (eg, ministries, educational institutes, the WHO, the Centers for Disease Control and Prevention [CDC], the United Nations Children’s Fund, and Médecins Sans Frontières) were involved in the development of 22% (5/23) of the analog games and 16% (7/43) of the digital games.

#### License

In total, 65% (15/23) of the analog games were commercial (price: US $6.54-33.77), 30% (7/23) were available for free (6/7, 86% of these as *Print & Play* games), and 9% (2/23) were Kickstarter prototypes. In contrast, the vast majority of digital games were free to play (20/43, 47%) or prototypes (20/43, 47%); 5% (2/43) were commercial (price: US $4.36-9.80), and 2% (1/43) were developed for internal use at a company.

#### Target Audience

Most games (38/66, 58%) targeted younger audiences, including children as young as 3 years old, teenagers, and university students. Analog games were often designed to be engaging for the entire family, whereas 36% (24/66) of all digital games did not have a defined target audience. Certain other games targeted very specific user groups, such as gamers, active social media users, health care workers, health enthusiasts, employees of certain enterprises, and the African population.

#### Game Types and Genres

Practically all games identified were simulations of the COVID-19 pandemic. Some were set in the players’ immediate environment, such as the classroom (eg, *Fighting COVID-19 at Purdue University*), the office (eg, *Social Distancing – The Game*), or the supermarket (eg, *Dino-Store*), or more broadly in a village, town, or city (eg, *Clinic Deluxe Edition: CoVid_19 variant* [Figure 1], *COVID Dodge, SurviveCovid-19,* and *VRS Fight Club*); a country (eg, *Better than Hugo* and *Korona hra*); or the world (eg, *Plague Inc.: The Cure*; Figure 2), where the players were tasked with controlling the spread of the disease. Others were set in a microscopic environment of the human body, where the players assumed the role of immune cells in fighting off the infection (eg, *Antidote COVID-19* [Figure 3] and *Infekcja*) or of the virus in spreading the disease (eg, *Viruscape*). Other interesting approaches were used in games such as *At-Home Scavenger Hunt* by the CDC (Figure 4)*,* which required the players to find relevant physical items in their actual environment; the location-based game *MeetDurian*, where virtual items could be collected in the player’s physical environment (similar to *Pokémon GO*) if the player was wearing a face mask, the presence of which was detected by face recognition software; and *GO VIRAL!* (Figure 5), which was set in the environment of a simulated social media platform.

The vast majority of analog games were board games (15/23, 65%) or card games (6/23, 26%). The most common analog game types or genres were strategy games (11/23, 48%; eg, *Lockdown!*; Figure 7) and race-to-the-end games (6/23, 26%; eg, *You Make Me Sick!*; Figure 6), whereas most digital games were primarily strategy (15/43, 35%; eg, *Plague Inc.: The Cure*), action (14/43, 33%; eg, *COVID Dodge*), and trivia (12/43, 28%; eg, *Corona Bee*; Figure 8) games. Some less common game types such as meditation or Zen (*CovidShield*), hidden object (*Help to stop the COVID-19 coronavirus* and *At-Home Scavenger Hunt* by the CDC)*,* memory (*Heroes of Covid-19*; Figure 9), and escape room–type puzzles (*COVID-19 [CORONA VIRUS]* and *Destroy COVID*; Figure 10) were also identified.

#### Deployment

Most digital games (23/43, 53%) were web-based and playable on PCs, mobile devices, consoles, or a combination of these. An additional 21% (9/43) were deployed for mobile platforms, 7% (3/43) were deployed for desktop and mobile platforms, 2% (1/43) were deployed for desktop only, 2% (1/43) were deployed for desktop and consoles, and 2% (1/43) were deployed for consoles only. Analog games mainly took the form of physical objects (eg, game boards with figures and dice, play money or tokens, playing cards, and puzzle pieces) or free electronic files that were designed to be printed at home (*Print & Play*).

#### Player Interaction

Of the 43 digital games, 37 (86%) were single-player, 4 (9%) were multiplayer (up to 7 players), and 2 (5%) were single- and multiplayer. Of the 6 multiplayer digital games, 5 (83%) were co-operative, and 1 (17%) was competitive. In contrast, all analog games (23/23, 100%) were multiplayer (up to 100 players), although single-player versions were also available in 26% (6/23) of the cases. Of these 23 games, 18 (78%) were competitive, 4 (17%) were co-operative, and 1 (4%) involved only teams or partnerships.

#### Gameplay Duration

Information retrieved from 15 analog games showed that gameplay duration ranged from 5 to 180 minutes—3 (20%) games required up to 15 minutes, 5 (33%) games required up to 30 minutes, 2 (13%) games required up to 45 minutes, 2 (13%) games required up to 60 minutes, and 3 (20%) games required >1 hour. The estimated duration could only be retrieved for 7% (3/43) of the digital games, which were substantially shorter than most analog games and lasted 5 to 15 minutes.

#### Intended Transfer Effects

##### Overview

In addition to a mostly entertaining *in-game goal,* serious games explicitly add a more *serious, out-game goal* [75], which refers to the intended effect of the game in the *real world* [28]; such an effect can be observed after playing the game and may include increased knowledge and awareness, skill development, and changes in attitudes or behaviors. Such serious games aim for impact that is typically long-term, in contrast to more short-term or direct forms of impact such as interactions, actions, and experiences while playing the game (for discussion on human impact–centered design, refer to the study by Fokkinga et al [77]). This repertoire of COVID-19 games covers this diversity of purposes, as summarized in Table 1 (extracted from the games’ descriptions; for details, refer to Multimedia Appendix 3).

**Table 1:** Intended transfer effects of COVID-19–themed games (listed according to the hierarchy of effects model originally developed for advertising and recommended by McGuire [78] for use in public health campaigns [79]; N=66).

Intended transfer effect		Games, n (%)	Examples
**Awareness-oriented effects**		11 (17)	
	Need for vaccination		Antidote COVID-19 Beat Corona Corona – Mit Eifer ins Geschäft COVID Challenge Heroes of Covid-19 Social Distancing – The Game
	Preventive measures		
	Need for well-being		
**Education-oriented effects**		38 (58)	
	Virus		Antidote COVID-19 Clinic Deluxe Edition: CoVid_19 variant Corona Bee COVID Safety Simulation: CAMPUS LIFE Govid You Make Me Sick!
	Policy		
	Vocabulary		
	Personal care		
	Information assessment		
**Attitude-oriented effects**		12 (18)	
	Improving empathy		Better than Hugo Breaking the Magic Circle Corona Game Essential Workers GO VIRAL! SurviveCovid-19++
	Sense of collective responsibility		
	Critical reflection		
**Behavior-oriented effects**		8 (12)	
	Executing preventive measures		At-Home Scavenger Hunt Corona Yuga CovidShield Game Suite Fighting COVID-19 at Purdue University MeetDurian The Magic Soldier of the Human Body
	Social conversation		
	Staying healthy		
	Entertainment		
	Maintaining well-being		

##### Increasing Awareness and Spreading Knowledge

Overall, the games reviewed in this study aim to educate the players on the COVID-19 pandemic in an entertaining way. Practically all games (66/66, 100%) were developed to spread general awareness about COVID-19 (eg, *Is COVID-19 caused by bacteria or a virus?*; *Name one symptom of COVID-19 that affects the respiratory system*; and *What does the “19” in COVID-19 stand for?*)*.* Some (6/66, 9%) were intended to educate people about the means and mechanisms of virus spread, the immune response, or both (eg, *True or False: COVID-19 can only be spread from person-to-person* and *Can you get COVID-19 from drinking water?*)*,* whereas others (34/66, 52%) focus more on safety precautions to avoid catching and spreading the infection (eg, *True or False: Wearing a face mask can help stop the spread of COVID-19 to others*; *When should you wash your hands?*; and *What is the minimum percentage of alcohol that a hand sanitizer must have?*)*.* Still others (5/66, 8%) specifically address vaccination (eg, *True or False: The COVID-19 vaccines can give you COVID-19*; *True or False: The vaccine will damage my DNA*; *I’m healthy and I’m never ill, should I still get vaccinated?*; and *Should a pregnant woman have the vaccine?*).

##### Influencing Attitudes

Several games (11/66, 17%) aim to cultivate people’s sense of empathy and collective responsibility (eg, *Clinic Deluxe Edition: CoVid_19 variant, Fighting COVID-19 at Purdue University,* and *Essential Workers*) as well as encourage critical reflection on policy makers’ dilemmas (eg, *Better than Hugo* and *Lockdown!*) or the spread of misinformation (eg, *CoronaChampion* and *GO VIRAL!*). *Heroes of Covid-19* may be the only game to focus on positive aspects of the pandemic, raising awareness of people’s noble deeds during this period (eg, *“*Quinn Callandar, a boy scout from Canada, used his 3D printing skills to create ear guards for people who feel pain from wearing masks all day*”*; *“*Intensive care nurse Molly Watts created a book entitled ‘Dave the Dog is worried about coronavirus’ to help tackle anxiety in children amid the Covid-19 outbreak*”*; and “Annemarie Plas from Brixton, London united the nation and gave the NHS frontline a boost with her ‘Clap for our Carers’ initiative.”).

##### Influencing Behavior

Some games were designed to promote adherence to safety precautions in real-life settings. For example, *Fighting COVID-19 at Purdue University* aims to encourage students to clean their university laboratories*,* and *MeetDurian* encourages players to wear protective masks in public. Other games aim to stimulate behavior that can directly improve the players’ well-being, such as mindfulness breathing (*CovidShield Game Suite*) and immunity-increasing nutrition (*The Magic Soldier of the Human Body*).

In most cases, the players were to adopt the positive role of a *hero* fighting the virus or protecting their avatar from infection in a simulated environment. An interesting yet less common alternative was to teach people about undesirable or unsafe behaviors by putting them in the negative role of, for example, the virus (*Viruscape*), an infected customer at a store (*Instructional remote multiplayer VR game*), or a misinformation spreader (*GO VIRAL!*).

### Testing of Effectiveness in Promoting Transfer Effects

A relatively small number of scientific studies that investigated the effectiveness of the individual COVID-19–themed games (20) have been published (Multimedia Appendix 4 [80-101]). In total, 3 categories of effects were most frequently tested: the change in participants’ knowledge of COVID-19 and infection prevention measures, their gameplay experience, and the games’ potential to facilitate players’ attitude or behavior change. The studies used various research methods and tools, including heuristic evaluations by experts, surveys composed of custom or standardized questionnaires and administered before or after gameplay, observations during gameplay, semistructured interviews, self-reports, or game log analysis after gameplay. A total of 15% (3/20) of the studies assessed the players’ changes in knowledge [80-82] and attitudes [80,83,84] by comparing their pre- and postgameplay responses. One study involved a control group that watched an educational video about COVID-19 for comparison with a test group that played the game [80], whereas another used comparative analysis with other similar games [85]. With the exception of 30% (6/20) of the studies, which involved larger numbers of participants or instances of gameplay, testing was performed on relatively small samples (2-30 participants).

At this point, it is important to emphasize the diversity of methods used to assess the effectiveness of the games as these can greatly limit the comparability of the results. Nevertheless, most of the studies considered (11/20, 55%) found that the games succeeded in engaging and motivating the players [80,81,83,84,87,91,94,99,101], increasing awareness, and facilitating teaching COVID-19 hygienic knowledge [80-83,89,91]. Improved understanding led to positive changes in players’ attitudes toward COVID-19 preventive measures [80,82,83,87,89,91,94,97,102]. A study reported that health anxiety remained relatively unchanged in response to playing 1 game [80].

Certain limitations of these studies have been acknowledged, especially in relation to the games’ capacity to change players’ behaviors. A proper assessment of the games’ capacity to influence behavior was found to be particularly challenging as different sources of information may have contributed to people’s adherence to safety measures [90,103] and as other factors—most notably, the players’ ages—also affected the games’ effectiveness [87,94]. The effectiveness of the reviewed games was often unclear or limited [98,103], and Suppan et al [92] explicitly stated that a longitudinal trial would be necessary to accurately assess their behavioral impact.

In addition, the studies recruited participants to play the games and evaluate their impact as opposed to surveying people who had already obtained and played the games through their own initiative. Information regarding the number of times the games were downloaded or played also does not appear to be structurally documented or made available; thus, it remains unclear to what extent the games were able to motivate people to play them in the first place. However, this information is of key importance as a game cannot be effective if it is not played.

## Discussion

### Principal Findings

This study aimed to provide an overview of games that were developed during the COVID-19 pandemic to disseminate crucial knowledge and enhance people’s subjective well-being. In general, we observed an extensive proliferation of games—especially digital games—in 2020, when the pandemic was declared. Most (31/66, 47%) were developed by research institutions in Europe and North America—some in collaboration with health authorities. All efforts notwithstanding, the overall frequency of use and impact of these games seem to have been modest at most.

To effectively influence people’s attitudes and behavior, design interventions need to be tailored to each situation, considering the environmental demands—as perceived by the target group—and their personal resources to meet these demands. On the basis of the findings of this study, we provide recommendations for the design of games aimed at improving people’s well-being in future global health crises similar to the COVID-19 pandemic.

### Game Development, Deployment, and Distribution

#### Overview

A game only has an impact if it is played. It is noteworthy that we only became aware of the multitude of COVID-19–themed games when we started actively searching for them. Although our experience is anecdotal, it does suggest the importance of investing time and effort in the dissemination of serious games.

#### Develop a Dissemination Plan

In the context of a pandemic, game developers should carefully consider their target audience. In many cases, this is probably the broadest audience possible, making it crucial to plan for the effective dissemination of the game. Most of the reviewed analog games (15/23, 65%) were commercially available physical objects, whereas most digital games (20/43, 47%) were freely accessible web-based applications. Each of the various forms of dissemination has advantages and disadvantages, but deployment strategy and cost may influence game accessibility to the greatest extent. Commercial games, for example, have an advantage in that they have access to professional dissemination channels and are often designed to be intriguing and esthetically appealing to attract people’s attention. However, the cost and effort necessary to obtain such games can preclude them from reaching a broader audience. For analog games, *Print & Play* games tend to be the most convenient to access, especially when freely available on the web. Such games have a relatively low threshold for access in that buying them is not necessary.

When social distancing is required, as was the case during the pandemic, playing analog games is typically only possible for members of the same household. In contrast, digital games are easy to obtain and play remotely provided that the users have access to electricity and the internet. To maximize reach, Hill et al [82] advised that COVID-19–themed games must be platform-independent and playable with minimal hardware.

Collaborating with health authorities, the media, nongovernmental organizations, educational institutions, (local) governments, and social media influencers can also help spread awareness of a game’s existence, although this may not always be the case. The designers of *Escape COVID-19*, for example, conducted a retrospective analysis to investigate the reasons for their limited success in recruiting participants for their study [104]. Specifically, they aimed to assess the effect of 3 different dissemination strategies on game account creation over a period of 6 months. In the first period (53 days), the game was disseminated by a part-time worker; following this was a press release (15 days); and in the final stage, the game was officially announced by the Swiss Federal Office of Public Health (15 days). Their findings suggested that the press release was the most successful and the official communication was the least successful. Nonetheless, the sequence and duration of communication interventions should also be considered.

#### Ensure the Reliability of Information

For games that aim to inform and share recommendations during a pandemic, a prominent challenge is that such recommendations are inherently dynamic. Although some information might be fixed (eg, what a virus is), other information may likely change after a game is introduced. The use of reliable sources such as the WHO, the CDC, and local governments can assure people that the communicated knowledge is factual. Practically all the reviewed games based their learning objectives on such sources. However, as guidelines may differ among different institutions and game developers may have different opinions on which information is correct, it is recommended that players be explicitly informed about the sources of the communicated knowledge. In addition, the credibility of the aforementioned sources can be subject to change, as seen with the rapidly waning trust in the CDC in the United States.

Real-time updates can be especially helpful in tackling confusion when guidelines change rapidly owing to the evolving understanding of health crises. It is critical that pandemic games be designed in a way that prevents them from becoming outdated too quickly; this can be addressed by incorporating sufficient flexibility in game design to allow for changing guidelines or information. Although this tends to be more challenging for analog games, it is possible—the content of analog games can be updated via expansion packs, and hybrid games where physical game elements are complemented by a regularly updated mobile app can be developed.

### Game Users, Design, and Use

How, where, when, and with whom a game is intended to be played can influence players’ motivation and the efficiency of the learning process [105]. Thus, it is important to design pandemic games that facilitate the acquisition and transfer of the intended learning objectives under pandemic-specific learning conditions.

#### Consider the Target Audience and Context of Use

##### Overview

When designing any serious game, it is generally advised to consider the entire ecosystem of stakeholders, including not only the players but also the initiators of games (eg, governments, nongovernmental institutions, health authorities, schools, and private citizens), design agencies, and game developers. The involvement of these stakeholders in various phases of game design (ie, problem definition, product design, and tailoring) can improve a game’s implementation [106]. For pandemic games, this can be challenging, especially if the game is intended to appeal to a broad variety of affected people. Therefore, it is necessary to determine exactly which stakeholders need to be targeted. The following sections focus specifically on player characteristics.

##### Player Demographics

According to the effectiveness studies we reviewed, certain target group characteristics—most notably, age—influence people’s engagement and the perceived effectiveness of the games [87,94]. Thus, caution is required when designing multiplayer games for people of different generations (eg, family games); it is important to avoid making such games too complex for very young or inexperienced audiences or too simple to effectively motivate adults and experienced players.

A general recommendation for game design is that game instructions should be easy to understand for a targeted user group. This recommendation is especially important for pandemic-focused games to minimize as much as possible the chances of spreading misinformation or confusion. Special attention needs to be paid to the language (preferably native language or multilingual versions) and vocabulary (preferably simple and familiar expressions) in the games. Using rules or elements from popular existing games (eg, *Monopoly, Ludo, Uno,* and *Pacman*) can help users understand and adopt a game more rapidly as people can only appreciate novelty when the product is also simultaneously perceived as familiar or typical [77,107].

##### Player Type

The process of meaning attribution can be influenced by the players’ culture, personality traits, and current mental state, as well as by other people [77]. Similarly, product emotions, which are the emotions users experience in response to (playing) the game, are influenced by their personal needs, goals, values, and abilities [77]. Thus, a detailed psychological understanding of a game’s target population can increase its impact.

First, it is important to understand that different people choose to play games for different reasons. A popular typology of player personalities developed by Bartle [108] distinguishes between 4 player types that differ based on their preferred way of engaging in the virtual world: *achievers, explorers, socializers,* and *killers* (for details, refer to the study by Bartle [108])*.* People assume different styles of play depending on their mood or in-game goals [108], suggesting that setting appropriate goals and using game elements to influence players’ moods may discourage adverse social in-game behavior. However, Bartle based his taxonomy specifically on multiuser dungeon games; thus, his taxonomy cannot be directly extrapolated to other game types. The player profiles by Bartle [108] might work for entertainment games but not for serious games. To address this issue, Siriaraya et al [28] advised that users’ experiential preferences be investigated for the game world as well as for the real world.

For COVID-19 games, it is also important to consider the differences between *social* and *solitary* players, especially in the degree of autonomy, presence, and relatedness they seek to experience (for details, refer to the study by Vella et al [109]). Understanding the specific needs and sources of motivation for different players can support the preferred player interaction.

#### Support Player Interaction

All the analog games reviewed in this study (23/23, 100%) were multiplayer and mainly competitive, whereas only 14% (6/43) of the digital games supported a multiplayer mode, of which 83% (5/6) were co-operative. The main advantage of single-player games is that they can be played at any time independently of other people. However, most single-player games do not allow for socializing, sharing opinions, or discussing ideas. In contrast, multiplayer games allow for socializing, but the minimum number of players required can limit the number of opportunities to play the game as this may depend on other people’s interest, availability, or both. Optimally, a game should be designed to support both single- and multiplayer modes, as was the case for 26% (6/23) of the analog games and 5% (2/43) of the digital games studied in this review.

During the COVID-19 pandemic, collective action and compliance with safety guidelines were paramount in reducing the spread of the virus. Co-operative games can communicate this need to the players and support social bonding and collective decision-making that can be transferred from the in-game world to real-life situations. Previous studies have suggested that participation in co-operative—as opposed to competitive—multiplayer games can support team building [110-113] and increase real-world collaboration and prosocial behaviors among players [114-117]. In contrast, the motivation to win in competitive games may leave little room for bonding and the exchange of ideas, possibly limiting the encouragement of prosocial behavior. Nevertheless, competitive games tend to be more stimulating, whereas co-operative games may support passive participation when responsibilities are not equally distributed among the players. Therefore, it is advised to design for teams or partnerships, which combines both co-operation among team members and competition against the opposing team or teams.

#### Balance Visual Design and the Realism of the Simulated Domain

Immersive simulations of realistic environments and experiences where acquired knowledge can be put to use without the serious consequences of a real disaster may facilitate players’ transfer of new knowledge to everyday life [29]; this is particularly important during pandemics, when rapid changes in attitudes and behavior are required. Presumably for this very reason, practically all the reviewed games were either literal or metaphorical simulations of the COVID-19 pandemic. However, as such games tend to emphasize educational goals, caution is necessary to adequately balance such education with entertainment so that games remain appealing to a broader audience.

To foster players’ motivation and consolidate their engagement, fun elements that are unrelated to the simulated domain (eg, avatar shape or a game within a game) can be used. For example, the authors of *Escape COVID-19* found that attractive graphics that were adapted to fit the preferences of the target population were essential in increasing players’ engagement [92]. Nevertheless, the designers of *Point of Contact* [82] stressed that a game’s graphics and cross-player communication need to be lightweight to minimize players’ dependence on high-performance devices and high-speed networks. Similarly, it is important to avoid substantial hardware demands, which were present in 2 game prototypes that were included in this study: the *Instructional remote multi-player VR game* requires access to a VR headset, and participants need to own an Xbox 360 device for the *Physical Fitness Training Program.*

#### Consider Time Requirements

Most of the analog games reviewed in this study (9/15, 60%) were designed for longer durations of gameplay (up to 3 hours); we could only retrieve the predicted duration for 7% (3/43) of the digital games, but the time required to play these games was considerably shorter (up to 15 minutes). The main advantage of short game durations (a few minutes) is that they do not require high levels of commitment, so the games can be played without previous planning. However, they may fail to elicit a *flow* state, reflection, or both. In contrast, long game durations (several hours) may deter potential users, especially if the game is multiplayer and requires scheduling a time that is appropriate for all players. Moreover, players may lose interest if a game lasts too long. Therefore, it is important to carefully design pandemic-themed games for optimal duration, depending heavily on the target group, game type, and intended transfer effects.

#### Challenge and Progression

To avoid cognitive overload, it is generally advised that games provide a clear distinction between the *active* and *reflective* phases of gameplay. Marne et al [105] suggested that intensive action phases that engage players emotionally and shift their focus toward a game’s goal can be used for practice and training, whereas less intensive phases should be provided for reflection and relief purposes. During reflective phases, games should provide feedback to players so that they can understand the consequences of their actions and keep track of their own progress. However, this information must be provided in a way that does not interfere with the player’s state of flow as this could cause them to lose interest in the game.

A possible solution proposed by Marne et al [105] is that individual items of obtainable knowledge or skills are represented as collectible virtual objects (*rewards*) that can be showcased or used as an asset later in the game. In addition to *material* rewards or achieving (publicly displayed) high scores, sensory stimulation (eg, sounds or graphics indicating an achievement) can also be rewarding, as can be messages of affirmation or commendation. For example, games such as *Escape COVID-19* included a positive message at the end of each level to strengthen players’ motivation to comply with guidelines [92]. In such cases, simple, commonly used terminology can help a game more effectively reach broader target audiences.

### Terminology for and Testing of Serious Games

In addition to the aforementioned considerations, our investigation revealed a need to agree upon a shared terminology and standardized testing approaches to improve the discourse on serious game development. From a scientific point of view, the wide diversity of findings at present renders it practically impossible to make any definitive conclusions or generalizations. A commonly agreed-upon method for effectiveness testing could facilitate the comparison of different approaches in game design and encourage the development of more impactful games. Consequently, we propose that a shared database of knowledge be created, such as the recently developed co.LAB methodological framework, which was implemented in a collaborative web platform that allows for the co-designing, codevelopment, and coevaluation of serious games [118].

### Limitations

Despite all the efforts to provide a comprehensive review of COVID-19–themed games, we acknowledge that other games that were not included in this report exist (eg, competition and game jam entries were excluded). Furthermore, information on games that were not described in scientific papers was obtained from various web-based sources, potentially limiting the study’s reliability. Nevertheless, we believe that this study correctly reflects the diversity of COVID-19–themed games.

### Conclusions

This study aimed to provide a structured overview of serious games developed in the context of the COVID-19 pandemic to improve people’s well-being through entertainment and education. We identified 66 diverse games, most of which were digital (43/66, 65%), were developed by research institutions in 2020 (13/66, 20%), and originated in Europe and North America (38/66, 58%). An analysis of the games’ characteristics was performed to identify potential pandemic-specific challenges and opportunities for improvement, and some recommendations were made based on the findings of the reviewed studies and existing game theories.

In total, 2 additional themes emerged as a result of this overview. First, better planning for effective dissemination of the games appears to be necessary if the goal of these games is to reach the broadest audience possible. Second, to increase the impact of similar future interventions, the collective effort of game developers and researchers is needed to advance the discourse on game design and testing.

## Appendix

Multimedia Appendix 1 Summary of analog COVID-19–themed games (N=23).

Multimedia Appendix 2 Summary of digital COVID-19–themed games (N=43).

Multimedia Appendix 3 Intended transfer effects of COVID-19–themed games.

Multimedia Appendix 4 Testing of COVID-19–themed games.

## Abbreviations

CDC: Centers for Disease Control and Prevention
WHO: World Health Organization
