# Supplementary material for: Games in Times of a Pandemic: Structured Overview of COVID-19 Serious Games
Source: JMIR Serious Games. 2023 Mar 7;11:e41766. doi: 10.2196/41766 (PMC9994467; doi:10.2196/41766)
Supplement: Multimedia Appendix 1 [file games_v11i1e41766_app1.doc]

**Multimedia Appendix 1.** Summary of analog COVID-19–themed games (N=23).

| **Game name** | **Release date** | **Country** | **Authorship** | **License** | **Deployment** | **Game type/genre** | **Target audience** | **Number**  **of players** | **Interplayer interaction** | **In-game goal** |
| --- | --- | --- | --- | --- | --- | --- | --- | --- | --- | --- |
| **At-Home Scavenger Hunt [47]** | September  2020 | — | CDC^a^ | Free  (“Print  & Play”) | Instruction printout | Scavenger hunt; adventure and search and identification (“Hidden Object”) | — | Multiplayer | Competitive, T/P^b^ | Collect the highest number of points by finding hidden items |
| **Beat Corona** | March  2020 | Belgium | Kaan Ilbas | Commercial  (US $14) | Cards | Card game; strategy (card shedding) | Aged  ≥7 years | Multiplayer  (3-9) | Competitive | Make pairs to get rid of all cards as quickly as possible; whoever is left with the corona card loses |
| **Beat Corona** | January  2021 | India | Grasp & Clasp | Commercial  (US $8) | Game board, figures, dice, playing cards, and coins | Board game; race to the end (“Snakes & Ladders”) | Aged  ≥5 years | Multiplayer  (2-4) | Competitive | Overcome obstacles, collect health coins, and reach the finish line first |
| **CDC board game: Protect Others & Protect Yourself from COVID-19 [48]** | November  2020 | — | CDC | Free  (“Print  & Play”) | Image printout | Board game; race to the end (“Snakes & Ladders”) | Aged  6-12 years | Single- and multiplayer  (1-4) | Competitive | — |
| **Clinic Deluxe Edition: CoVid_19 variant** | April  2020 | France | AV Studio Games | Commercial  (US $22) | Game board, figures, and tiles; web-based, desktop, and mobile version | Board game; survival and strategy (“Tile-laying”) | Aged  ≥16 years | Single- and multiplayer  (1-4) | Co-operative | Make decisions together, hire the best physicians, and build the best services to have the fewest deceased patients at the end |
| **Corona – Mit Eifer**  **ins Geschäft** | March  2020 | Germany | Sisters Schwaderlapp | Commercial  (US $27) | Game board, figures, dice, and playing cards | Board game; race to the end and survival | Aged  ≥7 years | Multiplayer  (2-4) | Competitive | Shop for your neighbor as quickly as possible without being infected |
| **Corona Battle Against Covid-19** | April  2020 | Germany | Markus Geiger, 3DARTLAB | Kickstarter prototype  (US $28-49) | Playing tokens | Board game; survival and strategy | — | Single- and multiplayer  (1-4) | Competitive | Collect 8 antidotes and survive the active turn to win |
| **Corona Kaartspel** | November  2020 | The Netherlands | Raad de Kaart | Commercial  (US $20) | Cards | Card game; trivia | Aged  12-65 years | Multiplayer  (2-12) | T/P | Collect points by guessing COVID-19–related words on the card in 30 seconds |
| **Corona Yuga** | January  2021 | India | Veer Kashyap, Teck Team Solutions | Commercial  (US $7) | Game board, figures, dice, and playing tokens | Board game; action and survival | Aged  ≥5 years | Multiplayer  (2-8) | Competitive | Complete all challenges successfully and survive COVID-19 |
| **COVID Survivor** | 2020 | — | Very Viral Games | Commercial  (US $15) | Playing cards, tokens, and dice | Card game; strategy and survival | Aged  ≥8 years | Multiplayer  (2-4) | Competitive | Fight off the infection and increase your chances of staying alive in the game |
| **Covid-19 Board Game**  **for Children** | August  2021 | South Africa | The Cape Winelands District Municipality | Free sets given  to the Western Cape Education Department | Game board, figures, and dice | Board game; race to the end (“Snakes & Ladders”) | Grade 2 students | Multiplayer | Competitive | — |

**Multimedia Appendix 1.** Summary of analog COVID-19–themed games (N=23; *continued*).

| **Game name** | **Release date** | **Country** | **Authorship** | **License** | **Deployment** | **Game type/genre** | **Target audience** | **Number**  **of players** | **Interplayer interaction** | **In-game goal** |
| --- | --- | --- | --- | --- | --- | --- | --- | --- | --- | --- |
| **COVID-19: A Race**  **to the Vaccine** | December  2020 | North Carolina, United States | Casey Blackert, Panther Creek  High School, and The Game Crafter | Commercial  (US $28) | Game board, figures, dice, and playing cards | Board game; action and strategy (“Ludo”) | Aged  ≥12 years | Multiplayer  (2-4) | Competitive | Bring all 4 pawns halfway around the board to get to the vaccine in the center while avoiding infected people |
| **Covidopoly19** | October  2020 | Connecticut, United States | Shahan Islam, CategoryTen | Commercial  (US $34) | Game board, figures, dice, playing cards, and play money | Board game; strategy (“Trading & Negotiation”) | Aged  ≥8 years | Multiplayer  (2-6) | Competitive | — |
| **Destroy COVID** | April  2020 | Hungary | Kristóf Horváth, Márton Geda | Commercial  (US $29) | Reusable puzzles similar to those in escape room games and a mobile app | Puzzle game; logic | Aged  ≥14 years | Multiplayer  (4) | Co-operative | Collaborate with coplayers to solve the puzzles and keep the number of infections to a minimum |
| **Help to stop the**  **COVID-19 coronavirus [49]** | 2020 | South Africa | UNICEF^c^  South Africa | Free  (“Print  & Play”) | Image printout | Board game; search and identification (“Hidden Object”) | Aged  ≥3 years | Single- and multiplayer | Co-operative | — |
| **Infected!** | May  2020 | Pennsylvania, United States | Sawyer and  Scott Alexander | Kickstarter prototype  (US $44) | Game board, playing cards, figures, and dice | Board game; race to the end and survival | — | Multiplayer  (2-4) | Competitive | Collect 3 keys to get your figures into the safe bunker |
| **Infection**  **(Infekcja) [50]** | May  2020 | Poland | University  of Gdansk | Free  (“Print  & Play”) | Game board, playing cards, and tokens | Board game; strategy and survival | — | Single- and multiplayer  (1-6) | Co-operative | Stimulate the immune system of all participants and fight off the coronavirus |
| **It’s contagious!** | November  2020 | California, United States | The Menagerie Collective | Commercial  (US $25) | Cards | Card game (card shedding) | Aged  ≥7 years | Multiplayer  (2-6) | Competitive | Avoid collecting cards by beating the value of the top card or using a power card to send the current viral load to your fellow players |
| **Lockdown!** | July  2021 | Singapore | National University of Singapore | Commercial  (US $17) | Cards | Card game; strategy | Aged  ≥7 years | Multiplayer  (2-6) | T/P | Implement various public health measures to save the most patients with COVID-19 in your country |
| **Pandemic Monopoly** | March  2020 | Australia | University  of Tasmania | Commercial | Game board, figures, dice, playing cards, and play money | Board game; strategy (“Trading & Negotiation”) | — | Multiplayer | Competitive | — |
| **Social Distancing –**  **The Game [51]** | April  2020 | India | DICE Toy Labs | Free  (“Print  & Play”) | Game board and instructions | Board game; strategy and logic | Aged  ≥5 years | Single- and multiplayer  (1-100) | Competitive | Position employees in the office and observe the spread of the virus; how many employees escaped the infection? |
| **The Lockdown** | 2020 | The Netherlands | Jessie De Jans | Commercial  (US $21) | Playing cards, tokens, and pawns | Card game; strategy and role-play | Aged  10-99 years | Multiplayer  (2-4) | Competitive | Assemble a team of specialists who can tackle this crisis as quickly and efficiently as possible, protect the right team members, and find the right balance between care and the economy to save the country |

**Multimedia Appendix 1.** Summary of analog COVID-19–themed games (N=23; *continued*).

| **Game name** | **Release date** | **Country** | **Authorship** | **License** | **Deployment** | **Game type/genre** | **Target audience** | **Number**  **of players** | **Interplayer interaction** | **In-game goal** |
| --- | --- | --- | --- | --- | --- | --- | --- | --- | --- | --- |
| **You Make Me Sick! [52]** | May  2020 | Pennsylvania, United States | Duquesne University | Free  (“Print  & Play”) | Game board, playing cards, and tokens | Board game; race to the end (“Snakes & Ladders”) | Aged  11-15 years | Multiplayer  (2-4) | Competitive | Collect immune cell tokens while traveling home from the hospital |

^a^CDC: Centers for Disease Control and Prevention.

^b^T/P: teams/partnerships.

^c^UNICEF: United Nations Children’s Fund.
