# Supplementary material for: Games in Times of a Pandemic: Structured Overview of COVID-19 Serious Games
Source: JMIR Serious Games. 2023 Mar 7;11:e41766. doi: 10.2196/41766 (PMC9994467; doi:10.2196/41766)
Supplement: Multimedia Appendix 2 [file games_v11i1e41766_app2.doc]

**Multimedia Appendix 2.** Summary of digital COVID-19–themed games (N=43).

| **Game name** | **Release date** | **Country** | **Authorship** | **License** | **Deployment** | **Game type/genre** | **Target audience** | **Number**  **of players** | **Interplayer interaction** | **In-game goal** |
| --- | --- | --- | --- | --- | --- | --- | --- | --- | --- | --- |
| **Adobe Flash–based game to educate children on COVID-19** | July  2020 | Indonesia | Universitas  Ngudi Waluyo | Prototype | — | Trivia;  “Drag-and-drop” | Children | Single-player | — | Match COVID-19–related expressions with pictures describing what to do, what to use, and when to go to hospital during the pandemic |
| **Antidote COVID-19 [53]** | October  2021 | Finland | Psyon Games  and the WHO^a^ | Free | Mobile | Simulation  and strategy  (“Tower defense”) | — | Single-player | — | Help the immune system fight off bacteria and viruses, including the coronavirus, and develop more and better vaccines |
| **Better than Hugo**  **(Beter dan Hugo) [54]** | January  2022 | The Netherlands | Quiten Coret | Free | Web-based | Simulation, strategy,  and role-play | — | Single-player | — | Introduce your own COVID-19 safety measures and try to do better than the Dutch government |
| **Breaking the Magic Circle** | October  2021 | England | University College London | Prototype | Web-based | Simulation  and role-play | University students | Single-player | — | Help an ICU^b^ nurse administer aid to patients with COVID-19 and save their lives |
| **Can You Save the World? [55]** | May  2020 | England | Richard Wiseman and Martin Jacob | Free | Web-based | Action  and adventure (“Runner” game) | Children | Single-player | — | Avoid close contact with others and maintain a safe distance while collecting personal protective equipment, toilet paper, and healthy food to gain more lives |
| **Corona Bee [56]** | March  2020 | Scotland | Focus Games | Free | Web-based | Trivia | — | Single-player | — | Collect points by answering COVID-19–related questions correctly |
| **Corona Game**  **(Korona hra) [57]** | January  2021 | Czech Republic | Michal Beneš,  Jan Walter,  and David Wagner | Free | Web-based | Simulation  and strategy | — | Single-player | — | Implement various safety measures in a country of 10 million citizens to minimize loss of life and economic impact within a predefined time frame |
| **CoronaChampion**  **(Fight against COVID-19) [58]** | September  2020 | India | UN^c^ Development Programme Accelerator Lab, New Delhi,  and IPE^f^ Global Centre for Knowledge and Development | Free | Mobile | Trivia | Youth and active social media users | Single-player | — | Assess whether 10 statements about COVID-19 are true or false and learn factual information |
| **CoronaQuest [59]** | May  2020 | Switzerland | Canton Vaud | Free | Web-based | Web-based  card game;  strategy | Aged 4-16 years | Single-player | — | Defend yourself against offensive card attacks using defensive cards |
| **COVID Safety Simulation: CAMPUS LIFE [60]** | September  2020 | Arizona,  United States | Arizona State University | Free | Web-based | Simulation  and trivia | College students | Single-player | — | Keep the probability of infection as low as possible by making correct choices |
| **COVID Challenge [61]** | November  2020 | Switzerland | Médecins Sans Frontières  and Pixel Impact | Free | Mobile | Trivia | — | Single-player | — | Correctly answer questions regarding a COVID-19–related scenario to collect points |
| **COVID Dodge** | March  2021 | Canada | Dalhousie University | Prototype | Desktop and mobile | Survival  and activity (“Runner” game) | The African population | Single-player | — | Avoid physical contact with oncoming villagers for a predetermined length of time |
| **COVID Pacman-C and**  **COVID Pacman-R** | July  2021 | Canada | Dalhousie University | Prototype | — | Survival  and activity  (“2D action maze”) | — | Single-player | — | Avoid getting close to the SARS-CoV-2 virus and other humans while collecting coins |
| **COVID-19 – Did You Know?** | April  2020 | Brazil | Federal University of Minas Gerais | Prototype | Mobile | Trivia | Teenagers | Single-player | — | Collect points by correctly answering questions regarding COVID-19 |

**Multimedia Appendix 2.** Summary of digital COVID-19–themed games (N=43; *continued*).

| **Game name** | **Release date** | **Country** | **Authorship** | **License** | **Deployment** | **Game type/genre** | **Target audience** | **Number**  **of players** | **Interplayer interaction** | **In-game goal** |
| --- | --- | --- | --- | --- | --- | --- | --- | --- | --- | --- |
| **COVID-19**  **(CORONA VIRUS)** | July  2020 | — | Insanity Game Studios | Commercial (US $4) | — | Simulation  and logic  (“Escape room”–type puzzle solving) | — | Single-player | — | Solve puzzles to escape quarantine |
| **Covid-19 Survivor** | 2021 | — | Different universities  in Greece, Brazil,  and Finland | Free for internal use | Web-based | Survival  and strategy | Middle school students | Single-player | — | Survive for as long as possible balancing the “Covid risk,” “money,” “physical,” “fun,” and “social” aspects and preventing them from crossing specific upper or lower limits |
| **COVID-Hero** | 2021 | Bangladesh | Science and Technology University | Prototype | Mobile | Action  and survival | Children | Single-player | — | Collect symbolic items such as “wearing a mask,” “washing hands,” and “maintaining distance” and avoid “crowd,” “virus,” and other harmful items to survive COVID-19 |
| **CovidShield Game Suite** | December  2020 | Portugal | Faculdade  de Motricidade Humana | Prototype | Mobile | Meditative/Zen | Aged ≥18 years | Single-player | — | Synchronize your breathing with the game through biofeedback to reduce tension and improve respiratory and cardiovascular function |
| **Dilemma Game –**  **Stay Safe Edition [62]** | July  2020 | Denmark | Lulu Lab | Free | Mobile | Simulation  and trivia | Aged 9-12 years | Single- and multiplayer | Co-operative | Solve dilemmas, ask questions, and obtain answers |
| **Dino-Store [63]** | October  2021 | Georgia, United States | Digital Integrative Liberal Arts Center, Georgia Institute  of Technology,  and Georgia Tech | Free | Web-based | Simulation  and activity  (“2D action maze” and “Stealth”) | — | Single-player | — | Navigate increasingly risky grocery stores with a limited view to successfully purchase items for your child’s birthday party; balance time against safety |
| **Escape COVID-19 [64]** | December  2020 | Switzerland and Portugal | University of Geneva Hospitals and Faculty of Medicine;  Geneva University Hospital;  and Hospital Garcia de Orta | Free | Web-based | Simulation,  trivia,  and role-play | Primarily health care workers and also the general public | Single-player | — | Choose adequate infection prevention and control behaviors for the scenario at hand to avoid COVID-19 |
| **Essential Workers** | November  2020 | Georgia, United States | Georgia Institute  of Technology | Prototype | Web-based | Simulation  and strategy | — | Multiplayer  (4) | Co-operative | Balance personal safety against economic necessities over the course of 3 weeks—choose between sheltering in place, earning a living, leaving home for essential supplies, and receiving medical services |
| **Fighting COVID-19**  **at Purdue University** | 2021 | Indiana,  United States | Purdue University | Prototype | Web-based | Simulation  and trivia | University students | Single-player | — | Follow the instructions for safe behavior in the classroom to avoid COVID-19 |
| **Go Corona Go [65]** | April  2021 | India | Abhinav Ranjith Das | Free | Web-based | Activity  (“Runner” game) | — | Single-player | — | Jump over obstacles and the virus, collect safety equipment to fight the virus, find the vaccine, and reach your final destination |
| **GO VIRAL! [66]** | May  2021 | England | University of Cambridge,  UK Cabinet Office, and WHO | Free | Web-based | Simulation, strategy,  and role-play | Aged ≥3 years | Single-player | — | Use manipulation strategies to maximize your likes and credibility on social media |
| **Govid** | March  2020 | India | Tata Consultancy Services | Free for internal use | Web-based | Survival  and trivia | Organization employees | Single-player | — | Answer questions related to COVID-19 dos and don’ts to feed the tiger, stay safe, and reach the shore |

**Multimedia Appendix 2.** Summary of digital COVID-19–themed games (N=43; *continued*).

| **Game name** | **Release date** | **Country** | **Authorship** | **License** | **Deployment** | **Game type/genre** | **Target audience** | **Number**  **of players** | **Interplayer interaction** | **In-game goal** |
| --- | --- | --- | --- | --- | --- | --- | --- | --- | --- | --- |
| **Heroes of Covid-19 [67]** | April  2020 | England | GRM Digital | Free | Web-based | Memory | — | Single-player | — | Match the faces of heroes in as few clicks as possible |
| **Infection Defender** | February  2021 | Denmark | COVID-19-CTRL GROUP,  Aalborg University | Prototype | Web-based | Simulation, strategy,  and action  (“Catch & Dodge”) | Aged 10-12 years | Single-player | — | Navigate the pandemic for a set number of days without overwhelming the hospital or negatively affecting people’s well-being |
| **Infection Detective** | March  2021 | Denmark | COVID-19-CTRL GROUP,  Aalborg University | Prototype | — | Simulation, strategy,  and action  (“Catch & Dodge”) | Primary and lower secondary school students | Single-player | — | Find infected people and isolate them to minimize disease spread until a vaccine is found |
| **Instructional remote multiplayer VR^d^ game** | January  2021 | Morocco | University of Patras | Prototype | Web-based | Simulation  and survival | — | Multiplayer  (2) | Competitive | Supermarket health manager: protect the customers from being infected by keeping the facility clean and safe for a predefined time frame; infected customer: spread the virus to surfaces and other customers of the supermarket within the predefined time frame |
| **MeetDurian [68]** | April  2021 | China | Qingdao University | Free | Desktop and mobile | Location-based; adventure  and trivia | — | Multiplayer | Co-operative | Capture 6 virtual durians while wearing a face mask by walking closer to them or by correctly answering COVID-19–related questions |
| **Physical Fitness Training Program** | September  2020 | Saudi Arabia | Imam Abdulrahman bin Faisal University | Prototype | Console | Activity  and sport | University students | Single-player | — | — |
| **Plague Inc.: The Cure [69]** | March  2021 | England | Ndemic Creations, WHO,  CEPI^e^,  and GOARN^f^ | Commercial (free extension) | Desktop and mobile | Simulation  and strategy | Aged ≥10 years | Single-player | — | Save the world by balancing public health measures, research and development, and social and economic factors to keep the disease under control while maintaining public trust |
| **Point of Contact** | August  2021 | England | Lancaster University | Prototype | Web-based | Strategy,  survival,  and role-play | Aged 19-25 years | Multiplayer  (4-7) | Co-operative | Avoid and prevent the spread of COVID-19 at the office |
| **Project Hospital—**  **Department of**  **Infectious Diseases** | August  2020 | Czech Republic | Oxymoron Games | Commercial (extension; US $10) | Desktop | Simulation  and strategy | — | Single-player | — | Transfer your patients to an isolation ward and prevent a potential outbreak of infectious diseases, including COVID-19 |
| **SurviveCovid-19** | May  2020 | India | IIT^g^ Tirupati | Prototype | Web-based | Strategy  and survival | — | Single-player | — | Collect all the required groceries and medicines while navigating the city and reach home without being infected or infecting other people |
| **SurviveCovid-19++** | July  2021 | India | IIT Tirupati | Prototype | Web-based | Strategy,  survival,  and role-play (profession-based) | — | Single- and multiplayer  (1-4) | Co-operative | Collect the required number of profession-specific items and vaccines while using masks and sanitizers to protect yourself from being infected |
| **The Corona Fighters** | August  2020 | India | Ministry of Health and Family Welfare | Free | Web-based | Action | — | Single-player | — | Collect hygiene and immunity boosters and kill viruses to prevent the spread of COVID-19 |
| **The Magic Soldier**  **of the Human Body** | October  2020 | Morocco | Faculté Polydisciplinaire  de Taza | Prototype | — | Adventure, memory,  and fight  (“Tower defense”) | — | Single-player | — | Defeat the coronavirus before it spreads within the castle (human body) and attacks all families (organs) |

**Multimedia Appendix 2.** Summary of digital COVID-19–themed games (N=43; *continued*).

| **Game name** | **Release date** | **Country** | **Authorship** | **License** | **Deployment** | **Game type/genre** | **Target audience** | **Number**  **of players** | **Interplayer interaction** | **In-game goal** |
| --- | --- | --- | --- | --- | --- | --- | --- | --- | --- | --- |
| **Unus Terra** | 2020 | Finland | University of Oulu | Prototype | Mobile | Strategy  and survival | Aged 12-35 years | Single-player | — | Discover new pandemic threats to the world and prevent viruses from spreading by defeating them in matches at different locations |
| **Viruscape** | October  2021 | Massachusetts,  United States | Harvard University Graduate School  of Design | Prototype | Desktop and console | Simulation,  action,  and survival | Health enthusiasts and gamers | Single-player | — | Infect as many cells as possible to create more virus, debilitate the immune reaction, and spread to the next patient |
| **VRS Fight Club [70]** | March  2020 | Portugal | Magikbee | Free | Mobile | Survival  and activity (“Runner” game) | Aged ≥12 years | Single-player | — | Avoid becoming ill while traveling around global cities by following WHO recommendations and collect as many vaccines as possible to save the world from COVID-19 |
| **WSG-COVID-19.SP** | February  2021 | Taiwan | Different universities | Prototype | Web-based | Scenario-based game and trivia | — | Single-player | — | Make the correct behavioral choices to avoid COVID-19 and self-assess repeatedly to correct any misconceptions and problematic behaviors |

^a^WHO: World Health Organization.

^b^ICU: intensive care unit.

^c^UN: United Nations.

^d^VR: virtual reality.

^e^CEPI: Coalition for Epidemic Preparedness Innovations.

^f^GOARN: Global Outbreak Alert and Response Network.

^g^IIT: Indian Institute of Technology.
