# Supplementary material for: Games in Times of a Pandemic: Structured Overview of COVID-19 Serious Games
Source: JMIR Serious Games. 2023 Mar 7;11:e41766. doi: 10.2196/41766 (PMC9994467; doi:10.2196/41766)
Supplement: Multimedia Appendix 3 [file games_v11i1e41766_app3.doc]

**Multimedia Appendix 3.** Intended transfer effects of COVID-19-themed games.

| **Game** | **Intended transfer effect** |
| --- | --- |
|  | **SPREADING GENERAL AWARENESS** |
| **Antidote COVID-19** | To spread awareness of COVID-19, vaccinations, and other preventive measures and communicate the importance and urgency of taking preventive actions against COVID-19. |
| **Beat Corona** | To encourage conversation about COVID-19. |
| **Corona – Mit Eifer ins Geschäft** | To entertain and educate about COVID-19. |
| **Infected!** | To entertain and educate about COVID-19. |
| **COVID Challenge** | To increase awareness, prevent misinformation, and encourage changes in habits linked to the risk of coronavirus infection. |
| **Covid-19 Survivor** | To empower students’ awareness of the risks and consequences of the pandemic through decision-making and system analysis. |
| **COVID-Hero** | To teach and create awareness about COVID-19 among children. |
| **Go Corona Go** | To cheer people up during the lockdown and spread awareness about COVID-19. |
| **Heroes of Covid-19** | To raise awareness of the positive stories about coronavirus. |
| **Social Distancing – The Game** | To spread awareness about the spread of COVID-19 at the office. |
| **The Magic Soldier of the Human Body** | To raise awareness and train players to adopt good eating habits so that they can strengthen their immune system and fight COVID-19. |
|  | **IMPROVING KNOWLEDGE** |
|  | **Means and mechanisms of virus spread and the immune response** |
| **Antidote COVID-19** | To spread awareness about COVID-19, vaccinations, and other preventive measures to communicate the importance and urgency of taking preventive actions against COVID-19. |
| **Dino-Store** | To reflect on the difficulties and anxieties of COVID-19 and help people understand how COVID can spread in public spaces to ultimately change people’s attitudes and behavior during the pandemic. |
| **Infection** | To familiarize players with the subject of COVID-19 and educate them about the immune system. |
| **Instructional remote multiplayer**  **VR game** | To familiarize with and inform about the coronavirus’s methods of transmission. |
| **Plague Inc.: The Cure** | To educate people on how to spot and stop a pandemic. |
| **Viruscape** | To promote a more systematic learning of human health concepts and coronavirus mechanisms, as well as advance medical literacy for the general public. |
|  | **Preventive measures and safety precautions** |
| **Adobe Flash-Based Game to Educate Children about Covid-19** | To teach children English vocabulary related to COVID-19 and safe behavior during the pandemic, as well as help them comprehend the policies established in relation to the outbreak. |
| **Antidote COVID-19** | To spread awareness about COVID-19, vaccinations, and other preventive measures and ultimately communicate the importance and urgency of taking preventive actions against COVID-19. |
| **Breaking the Magic Circle** | To persuade people to adhere to COVID-19 safety guidelines and build their empathy toward medical staff during the pandemic. |
| **Can You Save the World?** | To help children understand how social distancing can save lives during the coronavirus pandemic. |
| **CDC Board Game: Protect Others & Protect Yourself from COVID-19** | To teach players how to protect others and themselves from COVID-19. |
| **CoronaQuest** | To inform pupils about protective measures and actions that can make their return to school less stressful and safer and encourage them to foster more caring attitudes toward each other. |
| **COVID Dodge** | To raise awareness about social distancing and other precautionary measures against the spread of COVID-19. |
| **COVID Pacman-C; COVID Pacman-R** | To motivate people to adhere to COVID-19 precautionary measures. |
| **COVID Safety Simulation: CAMPUS LIFE** | To affect behavioral changes in college-aged students during the COVID-19 pandemic. |
| **COVID Survivor** | To teach players in a “good-humored” way any pandemic protocols that might sustain real actions that can bring about greater protection from future pandemics. |
| **COVID-19 – Did You Know?** | To provide science-based information on the prevention of COVID-19 and personal care during the pandemic while assessing players’ knowledge of COVID-19–related topics. |
| **Covid-19 board game for children** | To teach children safety measures needed to fight COVID-19 and keep their teachers and parents safe from infection so that schools can remain open and those who care for children stay healthy and continue working. |
| **Dilemma Game**  **– Stay Safe Edition** | To empower, educate, and engage users in learning about personal hygiene, social distancing, and safety precautions. |
| **Escape COVID-19** | To teach COVID-19 infection prevention and control practices. |
| **Fighting COVID-19 at Purdue University** | To teach best hygienic practices for preventing COVID-19 and mitigating its spread. |
| **Govid** | To address misinformation and the associated panic regarding COVID-19 dos and don’ts, as well as articulate certain policies that the organization’s employees should follow. |
| **Help to stop the COVID-19 coronavirus** | To give parents a chance to talk to their children about the virus and help them learn about how to keep healthy and safe in a fun way. |
| **Infection Defender** | To promote children’s understanding of closing schools, social distancing, testing, and hospitalization and ultimately fight the spread of infectious diseases in Denmark. |
| **Infection Detective** | To explain to primary school children the exponential growth in disease spread and how tests and isolation can stop contagious diseases. |
| **Lockdown!** | To raise public awareness and understanding of COVID-19 public health measures. |
| **MeetDurian** | To improve users’ hygiene habits and reduce virus dispersal. |
| **Point of Contact** | To change participants’ perceptions of COVID-19 preventive measures. |
| **SurviveCovid-19** | To help people understand the importance of masks, sanitizers, and social distancing and keep themselves safe from COVID-19 in a city. |
| **The Corona Fighters** | To influence players' actions in the real world by teaching them the right tools and behaviors to fight the COVID-19 pandemic and remind them to take the appropriate precautions and avoid infection. |
| **Unus Terra** | To encourage social distancing among adolescents and young adults. |
| **VRS Fight Club** | To bring fun while still reinforcing important messages about how to avoid virus spread. |
| **WSG-COVID-19.SP** | To promote effective learning strategies for protecting oneself against COVID-19. |
|  | **Vaccination** |
| **Antidote COVID-19** | To spread awareness about COVID-19, vaccinations, and other preventive measures to communicate the importance and urgency of taking preventive actions against COVID-19. |
| **Clinic Deluxe Edition: CoVid_19 variant** | To support researchers around the world in finding a COVID-19 vaccine in the near future. |
| **Corona Bee** | To increase COVID-19 vaccine uptake by challenging the myths and misconceptions that stop people from being vaccinated. |
| **COVID-19: A Race to the Vaccine** | To bring families together on the quest for a vaccine. |
| **You Make Me Sick!** | To reduce anxiety about COVID-19 and combat vaccine misinformation for children, parents, and teachers. |
|  | **INFLUENCING ATTITUDES AND BEHAVIORS** |
|  | **Building empathy and collective responsibility** |
| **Beat Corona** | To spread the message that human strength lies in being aware and united. |
| **Breaking the Magic Circle** | To persuade people to adhere to COVID-19 safety guidelines and build their empathy toward medical staff during the pandemic. |
| **Covid-19 board game for children** | To teach children safety measures needed to fight COVID-19 and keep their teachers and parents safe from infection so that schools can remain open and those who care for children stay healthy and continue working. |
| **Covidopoly19** | To encourage people to learn and have fun, as well as stimulate discussion about the novel coronavirus pandemic and social responsibility. |
| **Destroy COVID** | To enable people to play a board game while physically distanced, as well as communicate and collaborate effectively. |
| **Essential Workers** | To encourage players to reflect on how one person’s actions can affect other players in their simulated community and emphasize the need for community cooperation in stopping COVID-19. |
| **SurviveCovid-19++** | To educate people about the responsibilities of citizens and emphasize the need for collaboration in following safety measures to fight the pandemic. |
|  | **Encouraging critical reflection** |
| **Better than Hugo** | To provide people criticizing the government with an opportunity to experience the difficulties of COVID-19-related decision-making. |
| **Corona Game** | To build basic quantitative intuition about the behavior of the epidemiological model that the general public and decision makers might lack. |
| **CoronaChampion**  **(Fight against COVID-19)** | To tackle the surge of COVID-19 misinformation and fake news regarding COVID-19. |
| **GO VIRAL!** | To preemptively debunk misinformation regarding COVID-19 by exposing players to the techniques and motivations behind the spread of fake news on social media and “inoculate” them against its influence. |
|  | **IMPROVING WELLBEING** |
| **At-Home Scavenger Hunt** | To promote conversation about staying physically, emotionally, and mentally healthy during COVID-19. |
| **Corona Yuga** | To teach children about coronavirus protocols and encourage them to have fun during lockdowns. |
| **CovidShield Game Suite** | To improve the quality of players’ lives through mindfulness breathing practice. |
| **Physical Fitness Training Program** | To foster psychological health through a physical fitness training program among university students during the COVID-19 pandemic. |
