# Supplementary material for: Games in Times of a Pandemic: Structured Overview of COVID-19 Serious Games
Source: JMIR Serious Games. 2023 Mar 7;11:e41766. doi: 10.2196/41766 (PMC9994467; doi:10.2196/41766)
Supplement: Multimedia Appendix 4 [file games_v11i1e41766_app4.doc]

**Multimedia Appendix 4.** Testing of COVID-19–themed games.

| Study | Tested effect | | | Test(s) used | Participants, N | Timing  of testing | | | |
| --- | --- | --- | --- | --- | --- | --- | --- | --- | --- |
|  | COVID-19 knowledge | Gameplay experience | Attitude or behavior change |  |  | Before gameplay | During gameplay | Directly after gameplay | Long-term effects |
| Nurjanah et al [86] |  | ✓ |  | - Questionnaire (5-point Likert scale):   - Content appropriateness   - Language and understandability   - Game layout (placement of elements, colors, and font)   - Ease of use | 28 mothers of second-grade children and  5 English teachers |  |  | ✓ |  |
| Holliday [83] |  |  | ✓ | - Attitude measures (questionnaire: 10-point scales of agreement with statements related to the game’s goals) | 11 (some students) | ✓ |  | ✓ | ✓ |
|  |  |  | ✓ | - Qualitative feedback | 11 (some students) |  |  | ✓ |  |
|  |  |  | ✓ | - The “lingering effect”:   - Memories of the game   - Sharing or discussion of the game with others   - Self-reported attitude or behavior change | 11 (some students) |  |  |  | ✓ |
| Mulchandani and Orji [87] |  | ✓ | ✓ | - Questionnaire to assess the perceived persuasiveness of the game (7-point Likert scale adapted from  Thomas et al [88]) | 131 |  |  | ✓ |  |
|  |  | ✓ | ✓ | - Semistructured interviews | 18 |  |  | ✓ |  |
| Grizioti et al [89] | ✓ |  | ✓ | - Game log file analysis | 26 high school students (mean age 13 years) |  |  | ✓ |  |
|  |  | ✓ | ✓ | - Observation and talk-aloud during gameplay | 26 high school students (mean age 13 years) |  | ✓ |  |  |
|  |  | ✓ | ✓ | - Semistructured interviews after 45 minutes of gameplay | 26 high school students (mean age 13 years) |  |  | ✓ |  |
| Gaspar et al [90] | ✓ |  |  | - Google Analytics weekly reports: analysis of the numbers of correct and incorrect answers per gameplay | 17,571 instances of gameplay |  | ✓ |  |  |
| Satu et al [91] |  | ✓ |  | - Web-based survey (5 questions; 5-point Likert scale): usefulness of this type of game | Parents, adolescents, adults, and mobile game experts | ✓ |  |  |  |
|  |  | ✓ |  | - Web-based survey after watching a video clip of the game (8 questions; 5-point Likert scale): user reactions | Parents, adolescents, adults, and mobile game experts |  |  | ✓ |  |
| Suppan et al [92] |  | ✓ |  | - Heuristic evaluation as proposed by Davids et al [93] | Game developers and nonclinical hospital staff |  | ✓ |  |  |
| Suppan et al [94] |  | ✓ | ✓ | - Web-based survey (5-point Likert scale):   - “This serious game is engaging, meaningful, useful, boring”   - “I will recommend this serious game to others” - Self-report about change in infection prevention practices (5-point Likert scale) | 1104 |  |  | ✓ |  |
| Anupam et al [95] |  | ✓ |  | - Observation during gameplay | Play testers |  | ✓ | ✓ |  |

**Multimedia Appendix 4.** Testing of COVID-19–themed games *(continued)*.

| Study | Tested effect | | | Test(s) used | Participants, N | Timing  of testing | | | |
| --- | --- | --- | --- | --- | --- | --- | --- | --- | --- |
|  | COVID-19 knowledge | Gameplay experience | Attitude or behavior change |  |  | Before gameplay | During gameplay | Directly after gameplay | Long-term effects |
| Kao et al [80] | ✓ |  |  | - Survey (comparison of 2 groups—playing the game or watching a video):   - PHCK^a^   - SWCK^b^   - COVID-19 Hygienic Workstation Self-Efficacy | 11 students; 138 web-based survey respondents | ✓ |  | ✓ | ✓ |
|  |  | ✓ |  | - Survey (comparison of 2 groups—playing the game or watching a video):   - IMI^c^ (subscales: interest/enjoyment, effort/importance, pressure/tension, and value/usefulness)   - PXI^d^ (game condition) or VES^e^ (video condition) | 11 students; 138 web-based survey |  |  | ✓ |  |
|  |  |  | ✓ | - Survey (comparison of 2 groups—playing the game and watching a video):   - COVID-19 Anxiety Questionnaire   - COVID-19 Positive Hygienic Attitude Questionnaire | 11 students; 138 web-based survey | ✓ |  | ✓ | ✓ |
|  |  |  | ✓ | - Observation: recordings of students’ cleaning behavior | 11 students |  |  | ✓ | ✓ |
| Chettoor Jayakrishnan  et al [81] | ✓ |  |  | - Survey—understanding of COVID-19–related dos and don’ts:   - Social distancing   - Organizational contacts   - Measures to break the COVID-19 chain   - COVID-19 vaccine | 2379 employees | ✓ |  | ✓ |  |
|  | ✓ |  |  | - Analysis of players’ in-game responses | 2379 employees |  | ✓ |  |  |
|  |  | ✓ |  | - Gameplay experience rating (5-point Likert scale):   - Engaging   - Educational   - Facilitates learning during gameplay | 2379 employees |  |  | ✓ |  |
| Nikolov and Madsen [96] |  | ✓ |  | - Think-aloud while/after testing the prototype | Students aged 10-13 years and teachers |  | ✓ |  |  |
|  |  | ✓ |  | - Web-based questionnaire:   - Gameplay experience (usability of menus, difficulty level, gameplay elements and feeling of the game, and clearness of the message)   - Play setup   - Maximum level reached | Students aged 10-13 years and teachers |  |  | ✓ |  |
| Chen et al [97] |  | ✓ | ✓ | - Survey:   - Game immersion   - User workload   - Social acceptance   - Learning outcomes   - Personal hygiene | N/A^f^ |  |  | ✓ |  |

**Multimedia Appendix 4.** Testing of COVID-19–themed games *(continued)*.

| Study | Tested effect | | | Test(s) used | Participants, N | Timing  of testing | | | |
| --- | --- | --- | --- | --- | --- | --- | --- | --- | --- |
|  | COVID-19 knowledge | Gameplay experience | Attitude or behavior change |  |  | Before gameplay | During gameplay | Directly after gameplay | Long-term effects |
| Jiang et al [98] |  | ✓ | ✓ | - Web-based structured-diary survey every second day:   - Gameplay time   - Emotions and mood changes   - Perception of the epidemic   - Game experience of the day   - Thoughts related to the epidemic | 26 students with no previous Plague, Inc. experience |  |  | ✓ |  |
| Hill et al [82] | ✓ |  |  | - List of preventive measures to select familiar ones and rank their importance on a 5-point Likert scale | 23 | ✓ |  | ✓ |  |
|  |  |  | ✓ | - Self-report perception questionnaire: reflective questions about the role of the player in the transmission of COVID-19 and whether the player’s actions were representative of how they behave in the real world - Analysis of game logs | 23 |  |  | ✓ |  |
| Venigalla et al [99] |  | ✓ |  | - Questionnaire based on MEEGA+^g^ (5-point Likert scale) | 30 |  |  | ✓ |  |
| Venigalla et al [84] |  |  | ✓ | - Questionnaire (5-point Likert scale):   - Perception of roles of individual professions   - Perception of the need for collaboration to control COVID-19 | 2 teams of 4 players | ✓ | ✓ | ✓ |  |
|  |  |  | ✓ | - Observation of behavior changes during 15-minute gameplay | 2 teams of 4 players |  | ✓ |  |  |
|  |  | ✓ | ✓ | - Questionnaire (5-point Likert scale):   - Attractiveness of game design   - Readability of fonts   - Meaningfulness of visual representation   - Attitude or behavior change   - Expectations regarding ease of use   - Level of challenge and motivation   - Amusement - Quality score based on MEEGA+ model | 2 teams of 4 players |  |  | ✓ |  |
| Bouroumane et al [85] |  | ✓ |  | - Comparative analysis with similar games (Corps Humain, Project Remedium, and CoronaQuest):   - Coherence between playful and serious aspect   - Motivational aspects   - Commitment, immersion, and quality of gaming experience | Game developers |  | ✓ |  |  |

**Multimedia Appendix 4.** Testing of COVID-19–themed games *(continued)*.

| Study | Tested effect | | | Test(s) used | Participants, N | Timing  of testing | | | |
| --- | --- | --- | --- | --- | --- | --- | --- | --- | --- |
|  | COVID-19 knowledge | Gameplay experience | Attitude or behavior change |  |  | Before gameplay | During gameplay | Directly after gameplay | Long-term effects |
| Pohjolainen et al [100] |  | ✓ |  | - Heuristic evaluation:   - Game usability   - Gameplay   - Mobility | 5 UX^h^ experts |  | ✓ |  |  |
|  |  | ✓ |  | - Observation during gameplay | 2 teenagers |  | ✓ |  |  |
|  |  | ✓ |  | - Semistructured interviews | 2 teenagers |  |  | ✓ |  |
| Su et al [101] |  | ✓ |  | - Survey based on the TAM^i^ (8 measurement scales; 5-point Likert scale):   - Perceived satisfaction   - Intention to use the game   - Perceived usefulness   - Perceived ease of use   - Perceived enjoyment - Comparison with conventional learning methods - Usefulness of feedback after gameplay - Cognitive load | 71 students |  |  | ✓ |  |

^a^PHCK: Public Health COVID-19 Knowledge.

^b^SWCK: Shared Workspace COVID-19 Knowledge.

^c^IMI: Intrinsic Motivation Inventory.

^d^PXI: Player Experience Inventory.

^e^VES: Video Experience Scale.

^f^N/A: not applicable.

^g^MEEGA+: Model for the Evaluation of Educational Games +.

^h^UX: user experience.

^i^TAM: technology acceptance model.
